# Supplementary material for: Indigenous Australian women's experiences of participation in cervical screening
Source: PLoS One. 2020 Jun 15;15(6):e0234536. doi: 10.1371/journal.pone.0234536 (PMC7295213; doi:10.1371/journal.pone.0234536)
Supplement: S1 Text — (DOCX) [file pone.0234536.s001.docx]

**S1 Text. Modified Screening Matters interview guide**


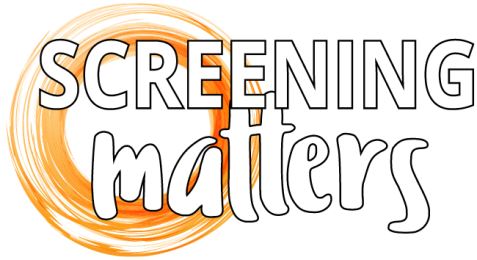
 **Screening matters: Aboriginal and Torres Strait Islander women’s attitudes and perspectives on participation in cervical screening**

**Yarning Guide – screened women**

**Please note the interview guide contains only the questions pertaining to the analysis reported in “Indigenous Australian women’s experiences of participation in cervical screening” by Butler et al.**

Thank you for coming today to talk with me/us about the cervical screening study. My name is ___ and this is ___ and we both work at Menzies School of Health Research in Brisbane.

We are very interested to hear from you about your experiences and opinions about cervical screening [this terminology changed to local language/reference as necessary].

There are no right or wrong answers – we are just interested to understand how you feel about screening and what your experiences of screening have been. Everything you tell us will be treated confidentially. Your name will not be used in any report or be given to the clinic staff. With your permission, we would like to record the interview so that we can concentrate on what you are saying and we will also write down some notes. Just reminding you that you are free to leave at any time without having to provide a reason.

**Knowledge about screening**

1. Have you heard much about these screening or pap tests?
2. Many people say that they don’t know much about cervical screening tests or pap tests or what they are for. Can you tell me what you understand this screening is for?
3. Where did you hear about these screening tests?
4. What sorts of things does the clinic tell you about screening tests?
5. How does the conversation with your doctor go about screening? What kinds of things do they say?
6. Have you heard other stories from friends or family about these tests? What sorts of things have you heard?

**Reasons for screening**

1. Can you tell me a bit about why you decided to have a screening test?
2. Can you tell me why you continue to screen? What encourages you?

**Views of screening**

1. Do you think that screening benefits you? In what ways?
2. How do you feel about having to see a doctor to be screened?

**Decision-making**

1. Did you know much about screening before you had your screening test?
2. What information helped you to decide to have the screening test?
3. Would you like more information about screening? Or do you feel you know enough?
4. Do you make the decision about having a screening test by yourself or do you like to talk about it with someone else?
5. Do other peoples’ views on whether you should have a test help you decide whether to do it or not?
6. If yes, in what ways? If no, why is that?

**Community views of screening**

1. Do you think that women in your community talk about screening at all?
2. Are there things that people in your community say about screening? Either good or bad?
3. What do you think about these things?
4. Do these views affect you wanting to have a screening test?
5. Is screening something you speak with other people in your community about?
6. If yes, what sorts of things do you tell other people about? What do you say?

**Experience with clinic**

1. Do you go to this clinic for all of your health needs?
2. Do you feel you have a good relationship with the doctor and other staff at this clinic?
3. What was experience of screening at this clinic? Were there parts of the process you were happy about? Were there parts you were unhappy about?
4. Can you tell me about any reminders you might receive when it is time to have a screen?
5. Are these reminders from the clinic useful?
6. Have you had any letters from the cervical screening program? Do you think these are useful?
7. Do these reminders encourage you to make an appointment or to have a screen straight away or the next time you attend the clinic?
8. Are there other ways that might help you to remember about booking a screening test?

**Suggestions that would make it easier for women to screen**

1. Some people have suggested that there could be a person at the clinic to help women navigate the screening process, from start to finish. This person would help women with screening and with follow-up after screening, and answer any questions women might have. What do you think about this idea?
2. Are there things that would make it easier for you or other women to get a screen test?

**Conclusion**

1. Do you have anything else you would like to say about screening today?
