# Supplementary material for: Indigenous Australian women's experiences of participation in cervical screening
Source: PLoS One. 2020 Jun 15;15(6):e0234536. doi: 10.1371/journal.pone.0234536 (PMC7295213; doi:10.1371/journal.pone.0234536)
Supplement: Attachment — Submitted filename: Response to reviewers.docx [file pone.0234536.s002.docx]

19 May 2020

Nelly Oelke

Academic Editor

PLOS ONE

Re: Article ID PONE-D-20-01555R1

Title: Indigenous Australian women's experiences of participation in cervical screening

Dear Nelly Oelke,

Thank you for comments on the revised manuscript raised in your email of 19 May 2020. Below we respond to your comments. Line references refer to the marked-up manuscript. In the revised manuscript, revisions are indicated using the Track Changes function of Microsoft word using the “show all revisions inline” setting.

Yours sincerely,

Dr Tamara Butler on behalf of the authors of PONE-D-20-01555R1

# Additional Editor Comments (if provided):

1. On page 9, Line 177, "understanding" should be "understand".

Revised as requested. See page 7, line 171. I also corrected an error in punctuation on line 170.

2. On page 10, Line 199, you state that you recruited both screened and unscreened women to participate. But the study reports only screened women were recruited or at the least that this data set only includes data from screened women. This needs to be clarified at the very least in the number of places where you differentiate between the two. But more so, if you have data from non-screened women, should these voices not also be reported?

To clarify this matter, I have moved the reference to unscreened women to page 7, line 163-165. This contextualises the recruitment of both screened and unscreened women in the larger Screening Matters study. I have also clarified terminology in this section by replacing “unscreened” women with “under-screened and never-screened” women in lines 160 and 164.

I have also added emphasis on the fact that only screened women’s views are reported in this paper on page 7, line 166-167, and this is also highlighted under Participants and Recruitment on page 8, line 176-177.

I searched for other references to unscreened women in the paper and could not find further places where unscreened women had been referenced, however I would be happy to clarify any other areas at your further instruction.

We decided to report the views of screened and under-screened/never-screened women’s views separately because strategies to enhance women’s participation in cervical screening differ greatly between those women who are already screening regularly and those who do not screen regularly. Furthermore, during the yarns we found that under-screened/never-screened women reported several issues that need to be reported with sensitivity and care (e.g., experiences of sexual trauma and discriminatory health care provision). Under-screened/never-screened women’s voices are rarely heard and are not well represented in the literature. We felt we could do the voices of both groups of women justice by separately reporting the experiences of each group. The manuscript for under-screened/never-screened women is currently in development.

3. On page 18, Line 314, "to be" should be changed to "for the."

Revised as requested. See page 14, line 274.

4. With regards to Table 2, I think it should be in the discussion as you suggest, but think that it would be better placed after the discussion of your analysed data. Discussions are generally written with the discussion of the data first, and then provide recommendations.

Revised as requested. Table 2 and text introducing the recommendations have been moved to after the discussion of findings. See page 29, line 550- page 33, line 555.
